# Application of Mathematical Modeling for Simulation and Analysis of Hypoplastic Left Heart Syndrome (HLHS) in Pre- and Postsurgery Conditions

**DOI:** 10.1155/2015/987293

**Published:** 2015-10-25

**Authors:** Ali Jalali, Gerard F. Jones, Daniel J. Licht, C. Nataraj

**Affiliations:** ^1^Department of Mechanical Engineering, Villanova University, Villanova, PA 19085, USA; ^2^Neurovascular Imaging Lab, University of Pennsylvania School of Medicine, Philadelphia, PA 19104, USA

## Abstract

This paper is concerned with the mathematical modeling of a severe and common congenital defect called hypoplastic left heart syndrome (HLHS). Surgical approaches are utilized for palliating this heart condition; however, a brain white matter injury called periventricular leukomalacia (PVL) occurs with high prevalence at or around the time of surgery, the exact cause of which is not known presently. Our main goal in this paper is to study the hemodynamic conditions under which HLHS physiology may lead to the occurrence of PVL. A lumped parameter model of the HLHS circulation has been developed integrating diffusion modeling of oxygen and carbon dioxide concentrations in order to study hemodynamic variables such as pressure, flow, and blood gas concentration. Results presented include calculations of blood pressures and flow rates in different parts of the circulation. Simulations also show changes in the ratio of pulmonary to systemic blood flow rates when the sizes of the patent ductus arteriosus and atrial septal defect are varied. These changes lead to unbalanced blood circulations and, when combined with low oxygen and carbon dioxide concentrations in arteries, result in poor oxygen delivery to the brain. We stipulate that PVL occurs as a consequence.

## 1. Introduction

**Figure 1:**
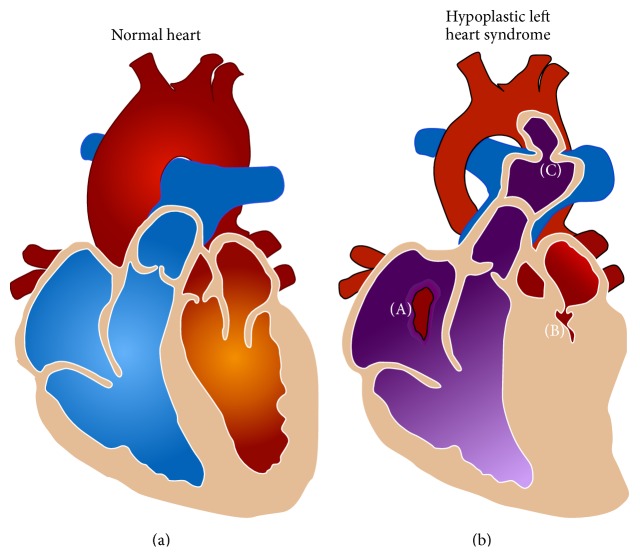
Comparison between normal heart on the left and HLHS heart on the right. (A) is atrial septal defect, (B) is hypoplastic heart, and (C) is patent ductus arteriosus. The left ventricle is very small. The aortic arch is extremely hypoplastic and flow is retrograde. Systemic output is ductal dependent. Adapted from wikipedia.

Hypoplastic left heart syndrome (HLHS) is a congenital heart defect (CHD) in which the left side of the heart is severely underdeveloped. Without early surgical palliation HLHS is universally fatal [1]. HLHS results from a failure of the aortic or mitral valve to form. Lack of antegrade flow through the valves causes insufficient growth of both the left ventricle and the ascending aortic arch. A typical HLHS heart is compared with a normal heart in Figure 1.

Underdevelopment of the left ventricle-aorta complex resulting in critical aortic valve stenosis or aortic valve atresia with an intact ventricular septum is the most recognized form of HLHS. There are corresponding changes in the right side of the heart in the case of HLHS. All right sided cardiac structures are larger than normal including the right atrium, pulmonary artery, and pulmonary valve [2].

As blood returns from the lungs to the left atrium, it must pass through an atrial septal defect to the right side of the heart. In cases of HLHS, the right side of the heart must pump blood to the body through a patent ductus arteriosus (PDA). This maintains fetal parallel circulation where the right ventricle is the only active pump [3]. But, since the ductus arteriosus (DA) usually closes within eleven days after birth, for an HLHS baby, blood flow is severely compromised leading to very low circulation and possible death. Hence, the management of neonates with HLHS is very complex. Treatment generally commences with vigorous infusion of prostaglandin to prevent the PDA from closing. However, reduction in pulmonary resistance after birth results in an unbalanced circulation where most of the blood goes into the pulmonary circulation thereby compromising the systemic oxygen supply. A typical way to treat this disease is shunt surgery. A shunt is a surgically created connection between the systemic arterial circulation and the pulmonary arteries. Authors in [4] present a good survey of shunt modeling and its application in planning the surgery.

The need for a detailed study of the problem of HLHS stems from our recent studies [5–7] to predict the occurrence and extent of periventricular leukomalacia (PVL) after Norwood surgery in neonates. The PVL is a form of white matter brain injury, characterized by necrosis (more often coagulation) of white matter near the lateral cerebral ventricles. It can affect newborns and (less commonly) fetuses; premature infants are at the greatest risk of the disorder. Affected individuals generally exhibit motor control problems or other developmental delays, and they often develop cerebral palsy or epilepsy later in life [8]. Despite being full-term infants, PVL is found in more than 50% of the neonates after cardiac surgery [1, 9]. As there has been a dramatic reduction in mortality rates following surgery for complex CHDs, there has been an increasing recognition of adverse neurodevelopmental sequelae in some survivors. Evaluation of children following neonatal repair of CHD demonstrates long term neurodevelopmental morbidity and learning disability in survivors of these infant heart surgeries. Management strategies before, during, and after surgery including the type of support during operation have been implicated as factors in postoperative neurodevelopmental dysfunction. Deficiency in oxygen circulation and low carbon dioxide concentrations (*P*_CO_2__) have also been considered as important factors affecting the morbidity rates of neurodevelopmental dysfunction [6].

In our previous work in the field of PVL prediction, we have applied computational intelligence techniques to the data obtained from 59 patients which were collected at the Children's Hospital of Philadelphia (CHOP). Computational intelligence (CI) is a set of nature-inspired computational methodologies, examples of which include Fuzzy Logic systems, Neural Networks, and Evolutionary Computation. The decision trees that we have developed suggest some ranges for critical hemodynamic parameters, such as oxygen concentration and blood pressure, which predict a high probability for the occurrence of PVL [5, 10, 11]. But since these numbers have been obtained from a retrospective review of limited set of subjects, they cannot be used as a general applicable criterion for all neonates. Hence, it is important to carry out physiological modeling of the cardiovascular system to begin to understand the underlying causal relationships between a particular set of parameters with the occurrence of PVL.

In this paper a lumped parameter model of the HLHS circulation has been developed to study blood pressure and flow in different parts of the cardiopulmonary circulation system. The diffusion modeling of oxygen (O_2_) and carbon dioxide (CO_2_) is also included in the model. Changes induced in O_2_ and CO_2_ concentrations and variation of blood flow rates in different parts of the body due to changes in some important model parameters have also been studied.

## 2. Pressure Flow Model

**Figure 2:**
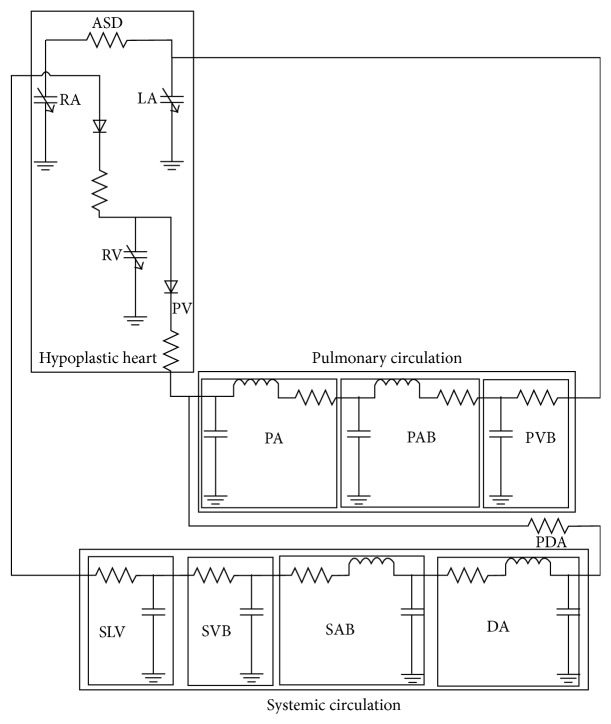
Lumped parameter model of HLHS. Model is made up of three parts: (a) hypoplastic left heart, (b) pulmonary circulation, and (c) systemic circulation. Direction of blood flow is shown by arrows. ASD: atrial septal defect; RA: right atrium; LA: left atrium; TV: tricuspid valve; RV: right ventricle; PV: pulmonary valve; PA: pulmonary artery; PAB: pulmonary arterial bed; PVB: pulmonary venous bed; PDA: patent ductus arteriosus; DA: descending aorta; SAB: systemic arterial bed; SVB: systemic venous bed; SLV: systemic large veins.

Several lumped parameter models of the cardiovascular system have been developed in past research, for instance, [12–14]. This paper differs from previously developed cardiovascular models in that this model is tailored to the unique physiology of the HLHS heart in comparison to the abundant literature which deals with the simulation of the normal heart. Furthermore, this model combines blood gas modeling with the circulation model to represent a more comprehensive model of the HLHS physiology. Moreover, by using parametric analysis in this study we have been able to track the changes in the heart for before and after surgery conditions. Like all other lumped parameter models, this model is limited by simplification assumption that it makes, for example, overlooking solid fluid interactions of arteries and blood flow and Newtonian flow assumption. For this work the lumped-parameter approach used to model the HLHS circulation is shown in Figure 2. Each box represents a lumped parameter model for a complex system of blood vessels and heart components. This model is built based on methodologies previously published for the fetal [15] and neonatal cerebral circulation [16–19] and the Norwood procedure [20, 21]. The complete model is composed of three main parts: a hypoplastic left heart, systemic circulation, and pulmonary circulation. Each compartment is made of resistances, capacitances, and inductances. Resistances are used to model the flow in the arteries and veins, and capacitors are used to represent the elasticity of these vessels. Inductors are not typically used for modeling the flow in the veins because veins do not function primarily in a contractile manner and so their inductance values are considered to be negligible in comparison with that of the arteries.

### 2.1. Hypoplastic Heart

The heart has been assumed to be composed of three parts: right atrium (RA), left atrium (LA), and right ventricle (RV). Here, as mentioned earlier, we have considered the most severe case of the hypoplastic heart in which the left ventricle is completely blocked (the most common form of HLHS is mitral atresia/aortic atresia; atresia implies no flow through the valve). Hence the left ventricle is not taken into consideration in the model.

The activity of the heart is modeled following the treatment in [22]. For both the atria and the ventricle, the total pressure is expressed as a nonlinear function of the volume and cycle-time:

(1) $$\begin{array}{c} P\left(t\right)=P_{s}\left(t\right)+P_{d}\left(t\right), \end{array}$$

where

(2) Ps=αtEmax⁡Vt−Vu,Pd=1−αtP0eKe·Vt−1.

*P* is the pressure, *V* is volume, *V*_*u*_ is the unstressed volume, and *E*_max⁡_ is the maximum elasticity of the heart wall during the heart cycle. The activation function *α* is the driving force of the heart and models the release of Ca^2+^ which initiates the contraction of the heart muscle [23].

**Figure 3:**
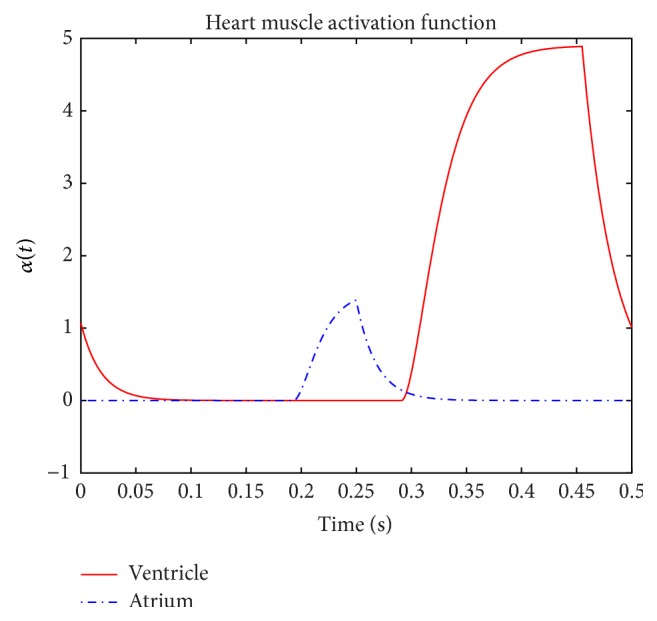
Ventricular and atrial activation functions.

Since the heart muscle's contraction is different for systole and diastole cycles, the activity function is defined by different differential equations in the systole and diastole. During diastole, the activation function is defined by the following:

(3) $$\begin{array}{c} \frac{d\alpha }{dt}=-K_{r}\alpha , \end{array}$$

where *K*_*r*_ is the relaxation rate. For the systole period it is defined by the following:

(4) $$\begin{array}{c} \frac{d^{2}\alpha }{d^{2}t}+2K_{e}\frac{d\alpha }{dt}+K_{e}^{2}\alpha =K_{e}^{2}\alpha_{\max}, \end{array}$$

where *K*_*e*_ is the excitation rate and *α*_max⁡_ is the limiting value of the activation function. The solutions to the above linear differential equations yield the following:

(5)  αt=αtde−Krt−td,αt=αmax⁡−αmax⁡−αts(1+Ke(t−ts))   ·e−Ket−ts,

where *t*_*s*_ is the systole time and *t*_*d*_ is the onset of diastole which we set equal to zero assuming the heart cycle starts from onset of diastole. This assumption is just for convenience and does not impact generalization of the model. The parameter values of *t*_*s*_, *K*_*e*_, *K*_*r*_, and *α*_max⁡_ are different for ventricles and atria and are shown in Table 1 [23]. The activation functions are functions of time for the ventricle and the atrium and are shown in Figure 3.

The *E*_max⁡_ is a function of volume of the ventricle to account for the decreasing elastance with increasing volume [20]. Consider

(6) $$\begin{array}{c} E_{\max}=E_{1}+E_{2}\left(V\left(t\right)-V_{u}\right), \end{array}$$

where *E*_1_ is constant; *E*_2_ is a negative-valued constant to account for decreasing elastance of the ventricle. *E*_2_ is zero for the atria resulting in a constant *E*_max⁡_, consistent with the literature. Again, *V*_*u*_ is the unstressed volume of the chamber which is the volume at zero pressure.

This is the *x*-intercept of the tangent at the end-systolic point for the pressure-volume (*P*-*V*) curve. For the diastole, the ventricle and atria fill through an exponential *P*-*V* function reflected in the equations below.

Hence, for maximum isometric pressure, we have [22]

(7) Pisomax,RAt=αnRA(t)E1,RA(VRA(t)−Vu,RA) +1−αnRAtP0,RAeKe·VRAt−1,Pisomax,LAt=αnLA(t)E1,LA(VLA(t)−Vu,LA) +1−αnLAtP0,LAeKe·VLAt−1,Pisomax,RVt=αnRVtE1,RV+E2,RVVRVt−Vu,RV ·VRVt−Vu,RV+1−αnRVtP0,RV ·eKe·VRVt−1,

where *α*_*n*_ is normalized *α*. The atria and ventricles are modeled with variable capacitors to account for the time-dependent relationship of pressure with volume and also viscous losses. A flow resistance has been introduced between the right and left atria to account for the defect in the walls of the heart permitting a mixing of blood flow between the atria. In the present work, this mixing has been modeled as an orifice unlike [24] where it was modeled as a simple resistor. The reason for modeling ASD as a variable nonlinear resistor instead of as a simple linear resistor is because of its very small diameter in comparison with other compartments which leads to a nonnegligible nonlinearity. A nonlinear pressure flow relationship (Darcy-Weisbach equation) is used as follows:

(8) $$\begin{array}{c} \Delta P\left(t\right)=K_{\text{ASD}}\cdot {Q\left(t\right)}^{2}. \end{array}$$

The tricuspid and the pulmonary valves are also modeled as unidirectional orifices (diodes) and a similar pressure flow relationship has been used for them. This model represents an idealized situation since, in reality, the tricuspid valve does leak sometimes and the flow is not completely unidirectional.

### 2.2. Pulmonary Circulation

The pulmonary circulation is divided into three compartments: proximal pulmonary arteries (PA), pulmonary arterial bed (PAB), and pulmonary venous bed (PVB). The PA and PAB are modeled using *RLC* and PVB by *RC* circuits. All these circuit elements have constant values. Flow enters into the PA from the pulmonary valve and the blood flows out to the left atrium (LA). It should be noted that the PDA is present in the newborn which normally closes after 5–10 days. To model it, a nonlinear resistor is added between the pulmonary artery and the aorta. The addition of the PDA to the model is another major difference between this model and other previously developed models; this is especially important in the current study because of our focus on HLHS.

### 2.3. Systemic Circulation

The systemic circulation is divided into four compartments: descending aorta (DA), systemic arterial bed (SAB), systemic venous bed (SVB), and systemic large veins (SLV). The DA and SAB are modeled by *RLC* and SVB and SLV are modeled by *RC* circuits. Blood comes in from the PDA and flows to the right atrium.

### 2.4. Fluid Flow

**Figure 4:**
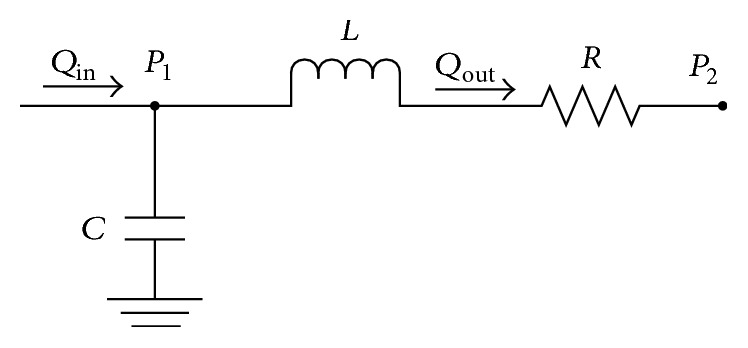
Simple RLC compartment to describe model. *P* is pressure; *L* (inductance) accounts for blood inertance; *R* (resistance) accounts for resistance to blood flow; *C* (capacitance) accounts for compliance.

For each compartment, time-dependent variables, such as pressure in the capacitance, are expressed as differential equations and since these equations cannot be solved analytically, numerical integration is used. This approach calculates solutions with variable time steps to avoid mathematical instability that would lead to divergence of the solution. For every RLC compartment shown in Figure 4, analysis has been carried out as shown.

The relationship between pressure and volume in the capacitor leg in Figure 4 can be calculated using the following:

(9) $$\begin{array}{c} dP\left(t\right)=\frac{dV\left(t\right)}{C}, \end{array}$$

where *dV*(*t*) is the change in the compartment volume calculated from the pressure differential. The pressure change *P*_1_ − *P*_2_ in Figure 4 is given by the following:

(10) $$\begin{array}{c} P_{1}\left(t\right)-P_{2}\left(t\right)=R\times Q_{\text{out}}+L\frac{dQ_{\text{out}}}{dt}. \end{array}$$

Hence, for every compartment, the change in the output flow rate is expressed using the following equation:

(11) $$\begin{array}{c} \frac{dQ_{\text{out}}}{dt}=\frac{P_{1}\left(t\right)-P_{2}\left(t\right)-Q_{\text{out}}\times R}{L}. \end{array}$$

ASD is assumed to be a variable nonlinear resistor; therefore, we can write the relationship between *Q* and pressure change through Darcy-Weisbach equation:

(12) $$\begin{array}{c} Q_{\text{ASD}}=\left\{\begin{array}{cc} \sqrt{\frac{P_{\text{RA}}-P_{\text{LA}}}{K_{\text{ASD}}}} & P_{\text{RA}}>P_{\text{LA}}\\ \sqrt{\frac{P_{\text{LA}}-P_{\text{RA}}}{K_{\text{ASD}}}} & P_{\text{LA}}>P_{\text{RA}}, \end{array}\right. \end{array}$$

where *P*_RA_ and *P*_LA_ are the right and left atrial pressures, respectively. By assuming realistic initial values, numerical integration is carried out for a sufficient number of heart cycles to achieve steady-periodic values for every parameter. The PDA flow rate is estimated from the following:

(13) $$\begin{array}{c} Q_{\text{PDA}}=\frac{P_{\text{PA}}-P_{\text{Aorta}}}{R_{\text{PDA}}}, \end{array}$$

where *P*_PA_ and *P*_Aorta_ are the pulmonary artery and descending aorta pressures, respectively.

### 2.5. Oxygen and Carbon Dioxide Diffusion Modeling in the Vascular System

The oxygen diffusion takes place from lungs to the alveolar capillaries and then from the blood capillaries to the body tissues. At steady state, the oxygen release and uptake will be equal, and hence we need to model only one diffusion process to find the partial pressures. The process of oxygen diffusion modeling is the same as what has been done in [20] but carbon dioxide calculation is added in our model. The reason for including CO_2_ diffusion to the model is that our previous findings have suggested blood CO_2_ concentration to be an important parameter in prediction of the PVL occurrence.

On the whole, the oxygen uptake and release equations can be written as was done in [25]. Consider

(14) $$\begin{array}{c} Q_{p}\left(C_{\text{pv}{\text{O}}_{2}}-C_{a{\text{O}}_{2}}\right)=\dot{\text{SV}{\text{O}}_{2}},\\ Q_{s}\left(C_{a{\text{O}}_{2}}-C_{v{\text{O}}_{2}}\right)=\dot{\text{CV}{\text{O}}_{2}},\\ \dot{\text{SV}{\text{O}}_{2}}=\dot{\text{CV}{\text{O}}_{2},} \end{array}$$

where $\dot{\text{SV}{\text{O}}_{2}}$ is the oxygen uptake rate in the lungs expressed in mL/min, and it is specified as an input to the system, CVO_2_ is the whole body oxygen consumption, *C*_pvO_2__ is oxygen concentration in the pulmonary vein, *C*_*a*O_2__ is oxygen concentration in the aorta, and *C*_*v*O_2__ is oxygen concentration in the veins. Since the PDA concentration of oxygen in aorta and pulmonary artery is equal, we simply replace oxygen content in the pulmonary artery with its value in the aorta in the second equation. It should be noted that this assumption is only true for the MA (atresia) patients.

The term *Q*_*p*_ is the average pulmonary flow obtained by taking an average of *Q*_ASD_ (atrial septal defect flows) over multiple cardiac cycles. There are two reasons. Firstly, under steady state conditions, all the pulmonary venous return flow passes through the ASD and pulmonary venous flow is equal to pulmonary flow. Hence, on average atrial septal defect flow and pulmonary flow are equal. Secondly, a large fraction of (ideally half) the pulmonary artery flow goes to aorta, and hence, to find the average pulmonary flow, it is more reliable to calculate *Q*_ASD_. Similarly, *Q*_*S*_ is the average systemic flow obtained by taking an average of PDA over the heart cycles.

Oxygen exchange in alveoli can be modeled using Hill's approach [26]. Oxygen in the blood is determined by the amount of oxygenated hemoglobin (98%) and the oxygen dissolved in blood (2%). So, the total volume of blood oxygen concentration (*C*_*b*O_2__) depends on the saturated blood oxygen (*S*_*b*O_2__) and the partial pressure of blood oxygen (*P*_*b*O_2__) by the model from [16]; this is shown in (15). The term *β* is the concentration of O_2_ in the blood hemoglobin at 100% saturation and the term *γ* is concentration of dissolved O_2_. *β* and *γ* have units of mL·mL^−1^mmHg^−1^ and the unit of concentration is mL/mL. One has

(15) $$\begin{array}{c} C_{b{\text{O}}_{2}}=\beta \cdot S_{b{\text{O}}_{2}}+\gamma \cdot P_{b{\text{O}}_{2}}. \end{array}$$

Differentiating (15), we get the following:

(16) $$\begin{array}{c} \frac{\partial C_{b{\text{O}}_{2}}}{\partial t}=\beta \cdot \frac{\partial S_{b{\text{O}}_{2}}}{\partial t}+\gamma \cdot \frac{\partial P_{b{\text{O}}_{2}}}{\partial t}. \end{array}$$

The relationship between oxygenized hemoglobin and dissolved oxygen is given by the oxygen dissociation curve:

(17) $$\begin{array}{c} \text{Hb}{\text{O}}_{2}=S_{b{\text{O}}_{2}}=\frac{{\left(P_{b{\text{O}}_{2}}/p50\right)}^{n}}{1+{\left(P_{b{\text{O}}_{2}}/p50\right)}^{n}}, \end{array}$$

where HbO_2_ is the oxygenized hemoglobin, *p*50 is the partial pressure of oxygen at 50% saturation (*S*_*b*O_2__ = 0.5), and *n* is a constant. Differentiating the above equation, we get the following:

(18) $$\begin{array}{c} \frac{\partial S_{b{\text{O}}_{2}}}{\partial t}=\left(\frac{\partial S_{b{\text{O}}_{2}}}{\partial P_{b{\text{O}}_{2}}}\right)\left(\frac{\partial P_{b{\text{O}}_{2}}}{\partial t}\right), \end{array}$$

where

(19) $$\begin{array}{c} \left(\frac{\partial S_{b{\text{O}}_{2}}}{\partial P_{b{\text{O}}_{2}}}\right)=\frac{1}{p50}\frac{n\times {\left(P_{b{\text{O}}_{2}}/p50\right)}^{n-1}}{{\left(1+{\left(P_{b{\text{O}}_{2}}/p50\right)}^{n}\right)}^{2}}. \end{array}$$

By combining the above equations, we have the following:

(20) $$\begin{array}{c} \frac{\partial C_{b{\text{O}}_{2}}}{\partial t}=\frac{\partial P_{b{\text{O}}_{2}}}{\partial t}\left(\gamma +\beta \left(\frac{\partial S_{b{\text{O}}_{2}}}{\partial P_{b{\text{O}}_{2}}}\right)\right). \end{array}$$

Assuming that oxygen in the capillaries of the lungs is at a constant partial pressure of 150 mmHg and that there is a continuous oxygen transfer from the lungs to the pulmonary blood capillaries, if we take the volume in the capillaries as a single unit with a volume *V*_cap_, then the time available for the transfer of oxygen to blood capillaries can be assumed to be the time taken for the pulmonary flow to fill the capillary volume, *V*_cap_. One has

(21) $$\begin{array}{c} t=\left(\frac{V_{\text{cap}}}{Q_{p}}\right), \end{array}$$

where *Q*_*p*_ is the pulmonary flow rate. At any point of time, the diffusion flux from the lungs to the blood capillaries is given by the following:

(22) $$\begin{array}{c} D_{{\text{O}}_{2}}=D_{L{\text{O}}_{2}}\left(P_{\text{alv}O_{2}}-P_{bO_{2}}\right), \end{array}$$

where *D*_*L*O_2__ is the total lung diffusion capacity for oxygen. The diffusion flux of oxygen *D*_O_2__ will lead to a change in the concentration of the volume in the blood capillaries, and hence we could write

(23) $$\begin{array}{c} D_{{\text{O}}_{2}}=V_{\text{cap}}\frac{\partial C_{b{\text{O}}_{2}}}{\partial t}. \end{array}$$

Thus, we have the following:

(24) $$\begin{array}{c} V_{\text{cap}}\frac{\partial C_{b{\text{O}}_{2}}}{\partial t}=D_{L{\text{O}}_{2}}\left(P_{\text{alv}{\text{O}}_{2}}-P_{b{\text{O}}_{2}}\right). \end{array}$$

Substituting for ∂*C*_*b*O_2__/∂*t* we get

(25) $$\begin{array}{c} V_{\text{cap}}\frac{\partial P_{b{\text{O}}_{2}}}{\partial t}\left(\gamma +\beta \left(\frac{\partial S_{b{\text{O}}_{2}}}{\partial P_{b{\text{O}}_{2}}}\right)\right)=D_{L{\text{O}}_{2}}\left(P_{\text{alv}{\text{O}}_{2}}-P_{b{\text{O}}_{2}}\right). \end{array}$$

Hence, at any instant, the change in the partial pressure of oxygen in blood capillaries by diffusion is given by the following:

(26) $$\begin{array}{c} \frac{\partial P_{b{\text{O}}_{2}}}{\partial t}=\frac{D_{L{\text{O}}_{2}}\left(P_{\text{alv}{\text{O}}_{2}}-P_{b{\text{O}}_{2}}\right)}{V_{\text{cap}}\left(\gamma +\beta \left(\partial S_{b{\text{O}}_{2}}/\partial P_{b{\text{O}}_{2}}\right)\right)}. \end{array}$$

Integrating this equation over the limits, by using the numerical trapezoidal integration method with a time step of 0.01 s,

(27) $$\begin{array}{c} \overset{P_{\text{pv}{\text{O}}_{2}}}{\underset{P_{a{\text{O}}_{2}}}{\int }}\frac{\left(\gamma +\beta \left(\partial S_{b{\text{O}}_{2}}/\partial P_{b{\text{O}}_{2}}\right)\right)}{\left(P_{\text{alv}{\text{O}}_{2}}-P_{b{\text{O}}_{2}}\right)}\;\partial P_{b{\text{O}}_{2}}=\frac{D_{L{\text{O}}_{2}}}{V_{\text{cap}}}\overset{V_{\text{cap}}/Q_{p}}{\underset{0}{\int }}\partial t, \end{array}$$

where *P*_pvO_2__ is the partial pressure of oxygen in the pulmonary veins. Since this is a complex function of *P*_*bO*_2__, integration is carried out numerically. An estimated value of *P*_*aO*_2__ is used as an initial guess and the value of *P*_pvO_2__ is calculated from the integral. If (27) is not satisfied, an improved value of *P*_*a*O_2__ is chosen and integration is repeated. This procedure is continued until (27) is satisfied to the required level of accuracy. The values of *P*_*a*O_2__ and *P*_pvO_2__ are checked to satisfy the integral equation and if the left hand side of (27) turns out to be less (or more) than the right hand side, then the integration is carried out again with a lower (or higher) value. This process is repeated until the equation is satisfied to the required level of accuracy. Using the final value of *P*_*a*O_2__ and oxygen release equation, the value of *P*_*v*O_2__ is calculated.

The exchange modeling for CO_2_ has been implemented using the same approach as we used for oxygen. The only difference is that the CO_2_ is released in the lungs and it is taken up by the blood from the body tissues. Hence, we have

(28) $$\begin{array}{c} Q_{p}\left(C_{a{\text{CO}}_{2}}-C_{\text{pv}{\text{CO}}_{2}}\right)=\dot{\text{SV}{\text{CO}}_{2}},\\ Q_{s}\left(C_{v{\text{CO}}_{2}}-C_{a{\text{CO}}_{2}}\right)=\dot{\text{CV}{\text{CO}}_{2}},\\ \dot{\text{SV}{\text{CO}}_{2}}=\dot{\text{CV}{\text{CO}}_{2}}, \end{array}$$

where $\dot{\text{SVC}{\text{O}}_{2}}$ is the CO_2_ release rate in the lungs expressed in mL/min, and it is specified as an input to the system, $\dot{\text{CVC}{\text{O}}_{2}}$ is the whole body CO_2_ release rate, *C*_pvCO_2__ is CO_2_ concentration in the pulmonary vein, *C*_*a*CO_2__ is CO_2_ concentration in the aorta, and *C*_*v*CO_2__ is CO_2_ concentration in the veins.

Referring to [17, 18] for the relationship of concentration of CO_2_ to its partial pressure at a temperature of 37°C and pH = 7.4 and neglecting the effect of oxygen saturation (which is reasonable), we get

(29) $$\begin{array}{c} C_{b{\text{CO}}_{2}}=0.009083P_{b{\text{CO}}_{2}}, \end{array}$$

(30) $$\begin{array}{c} \frac{\partial C_{b{\text{CO}}_{2}}}{\partial t}=0.009083\frac{\partial P_{b{\text{CO}}_{2}}}{\partial t}, \end{array}$$

where *C*_*b*CO_2__ is the blood CO_2_ concentration in mL/mL. The diffusion equation for CO_2_ becomes

(31) $$\begin{array}{c} V_{\text{cap}}\frac{\partial C_{b{\text{CO}}_{2}}}{\partial t}=D_{L{\text{CO}}_{2}}\left(P_{\text{alv}{\text{CO}}_{2}}-P_{b{\text{CO}}_{2}}\right), \end{array}$$

where *D*_*L*CO_2__ is diffusion capacity of lung for CO_2_ which is known and *P*_alvCO_2__ is alveolar pressure of CO_2_, which is set to 41 mmHg. Using (30) and (31), and after integration, we have

(32) $$\begin{array}{c} \overset{P_{\text{pv}{\text{CO}}_{2}}}{\underset{P_{a{\text{CO}}_{2}}}{\int }}\frac{0.009083}{\left(P_{\text{alvC}{\text{O}}_{2}}-P_{b{\text{CO}}_{2}}\right)}\;\partial P_{b{\text{O}}_{2}}=\frac{D_{L{\text{CO}}_{2}}}{V_{\text{cap}}}\overset{V_{\text{cap}}/Q_{p}}{\underset{0}{\int }}\partial t. \end{array}$$

Equation (32) is numerically integrated with an initial value of *P*_*a*CO_2__ such that the value of *P*_pvCO_2__ satisfies carbon dioxide release equation in the lungs. It is iterated until the equation is satisfied with a certain level of accuracy. Again, using the value of *P*_*a*CO_2__ and using the equation of carbon dioxide taken up by the blood, we get a solution for *P*_pvCO_2__.

### 2.6. Parameter Values

The parameter values for the case of a newborn HLHS patient were taken from a child after stage 1 circulation with a neoaorta and a Blalock-Taussig shunt replacing the PDA [20]. Since the first step Norwood procedure is applied for a child in his/her early few weeks, the parameter values for these infants would be comparable; these values are listed in Tables 1 [Table tab2]–3. It should be noted that *R*_ASD_, *R*_PDA_, and *R*_PA_ are the only variables which change postoperatively; we perform an analysis by varying the parameters to include a wide range of patients.

The value of the resistance for the pulmonary arteries is almost a hundred times that which is used in [20]. This is an estimated value to show very severe conditions. This is because, for a newborn baby, it is well known that pulmonary resistance is very high, falls precipitously after the first breath, and continues to fall over the first few weeks of his life. The average body surface area (BSA) for an adult male is around 1.5 m^2^ and the average surface area for a newborn child is 0.30 m^2^. Hence a scaling factor of five can be assumed while estimating some of the parameters for a child from the parameters of an adult. Although this method is widely used by clinicians in estimating the parameters, it brings some uncertainty to the model; hence, a parametric analysis has been carried out in order to understand the effect of those variables on the outputs.

The oxygen consumption rate in an average adult is 300 mL/min [24] and hence the average oxygen consumption rate in a child can be assumed to be approximately 60 mL/min. The total lung diffusion capacities for O_2_ and CO_2_ are 62 mL/min (mmHg)^−1^ and 478 mL/min (mmHg)^−1^, respectively, for an adult. Considering the scaling factor of 5 and the fact that the diffusion capacity is inversely proportional to the square of the surface area, we have a diffusion capacity for a child for O_2_ and CO_2_ as 2.5 mL/min (mmHg)^−1^ and 19 mL/min (mmHg)^−1^, respectively. Average CO_2_ release rate by an average adult is 400 mL/min, and hence the average release rate for a child is 80 mL/min.

### 2.7. Numerical Methods and Algorithms

The simulations and modeling presented in this paper have been done using Matlab. We used the trapezoidal integration method with a time step of 0.01 s for numerical integration. The numerical differentiation for solving ordinary differential equations presenting the HLHS model has been done using explicit fourth-order Runge-Kutta method [27]. In our simulations we have set the time step and time span to 0.01 s and 10 s, respectively. We have then ignored the first few cycles to drop the transients and to achieve steady-state solution. The initial condition has been set to 0.1 of the respective units for the pressures and flows. The computer used to run the simulations has a 32 GB of RAM and an Intel Xeon X5680 processor. The computation time to run the simulations for 10 s was 6.19 s.

## 3. Results

**Figure 5:**
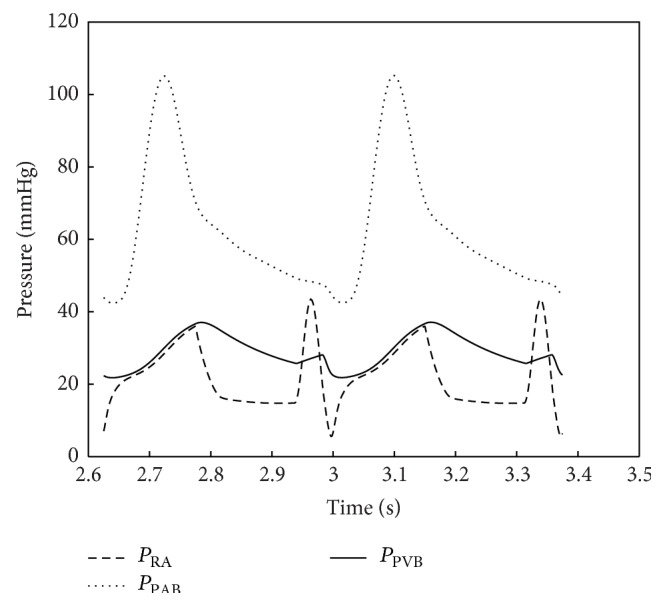
Pressures in right atrium, pulmonary artery, and pulmonary venous bed. As it is expected in the case of HLHS babies, since blood is pumped from right ventricle to the whole body, pressures in pulmonary arterial bed for the HLHS babies are higher than those for normal babies.

**Figure 6:**
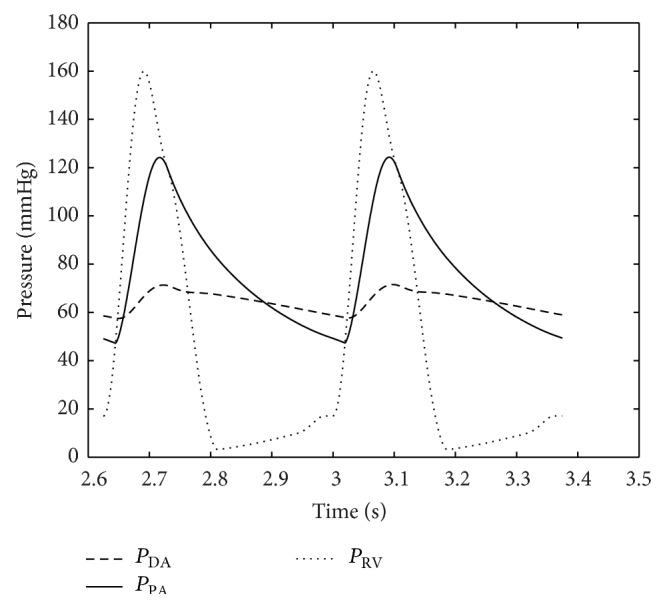
Pressures in right ventricle, descending aorta, and pulmonary artery. The heart rate is assumed to be 160 bpm based on the average HLHS patient data.

Numerical results were obtained using the parameter values as discussed above. Typical important pressure curves for various points of the body are shown in Figures 5 and 6. The pressures depicted by the model in Figures 5 and 6 are consistent with the atrial, ventricular, and pulmonary pressures of the HLHS heart. The pressure values of the different sections of the heart through the two contraction cycles are consistent with a heart that has a blockage/defect of the left ventricle as in patients with hypoplastic left heart syndrome, where the right ventricle is responsible for circulating blood to the lungs as well as throughout the body.

**Figure 7:**
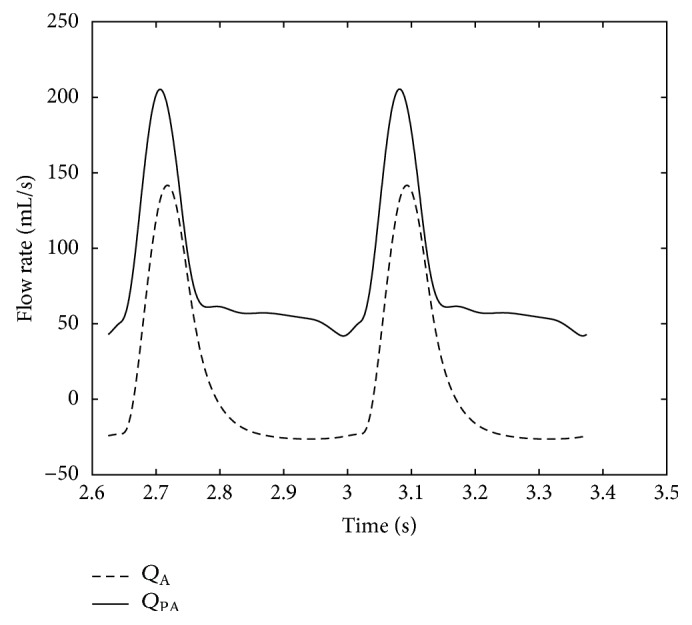
Flow rates in aorta and pulmonary artery. The plots are shown for *R*_ASD_ and *R*_PDA_ equal to 1 mmHg·s·mL^−1^. The neonatal situation shown in this figure is dangerous, because most of the flow goes to the pulmonary artery and there is not enough flow going to the vital organs.

**Figure 8:**
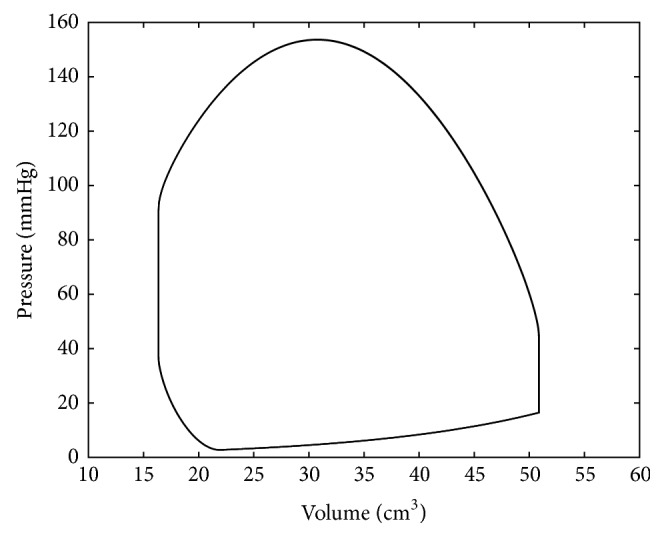
Pressure-volume loop. *R*_ASD_ and *R*_PDA_ are set to 0.1 mmHg·s·mL^−1^ and HR is 160 bpm.

The variations of flow rate in the aorta and the pulmonary artery are shown in Figure 7. Another very important curve is the *P*-*V* loop for the right ventricle, which is shown in Figure 8. There are no discontinuities or nonsmoothness in the figure since all the relationships that are used for calculating the pressures and the volumes are exponential. Note that the heart rate in this paper is assumed to be 160 bpm which is calculated from the mean values of patient data. The end-diastolic and systolic volumes for the right ventricle are 49.9 and 18.2 cm^3^, respectively, with an ejection fraction of 63.5%.

**Figure 9:**
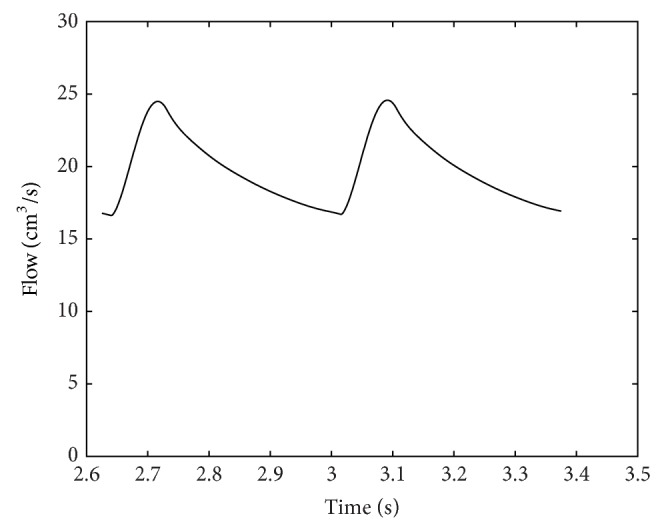
Flow rate in PDA for *R*_PDA_ is equal to 10 mmHg·s·mL^−1^.

Figure 9 displays the PDA flow rate. Increasing the resistance decreases the flow rate as might be expected.

### 3.1. Parametric Analysis

**Figure 10:**
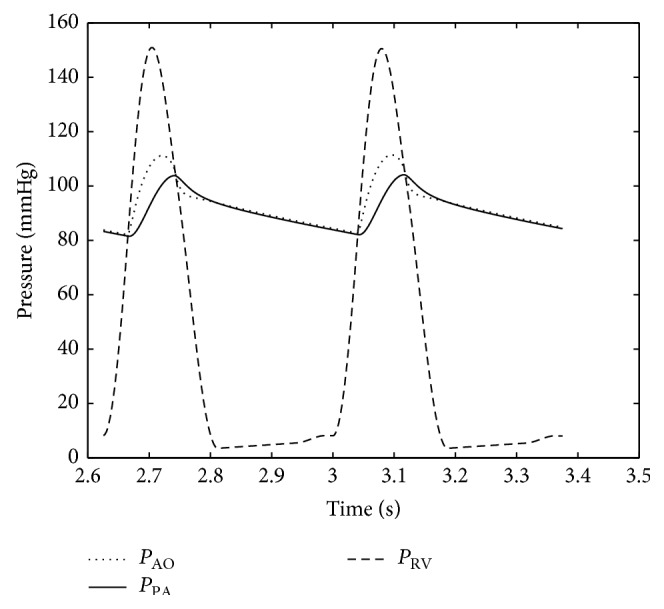
Pulmonary artery and aorta pressure just after birth in HLHS baby with *R*_PDA_ = 0.1 and *R*_PA_ = 2 mmHg·s·mL^−1^.

We performed a parametric analysis to investigate the effect of some clinically important parameters on the model performance and also to provide a way to compare different stages in the HLHS baby's life. When a baby is born, the PDA is fully open; hence *R*_PDA_ is low. When the baby takes his first breath, pulmonary resistance falls sharply in the first few hours but still remains at a high value for the first few days of life as it slowly drops down to normal. To model this phenomenon, we assume *R*_PDA_ = 0.1 and *R*_PA_ = 2 mmHg·s·mL^−1^; the resulting pressure curves for the different parts are as shown in Figure 10.

As can be seen in Figure 10, aortic pressures are in the normal range of 75–115 mmHg and the pulmonary pressures are also normal. But as the baby ages by 5–10 days, the PDA begins to close and hence *R*_PDA_ increases.

**Figure 11:**
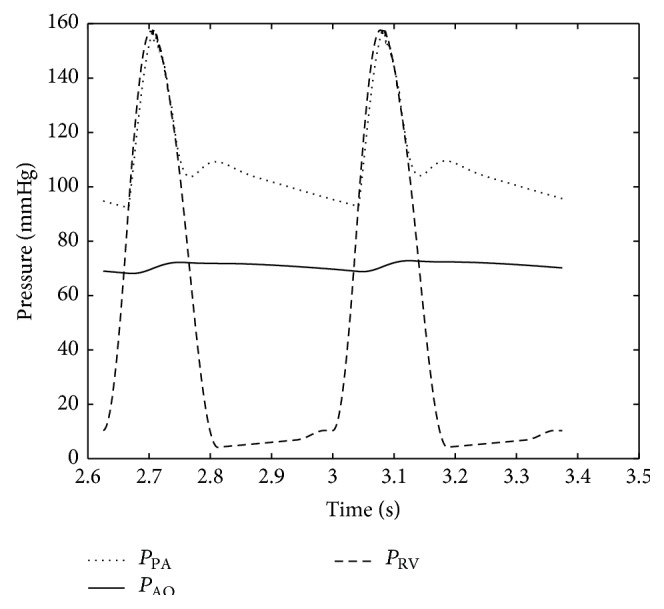
Pulmonary artery and aorta and right ventricle pressure before Norwood circulation procedure.

As the pulmonary resistance further drops to *R*_PA_ = 0.2 mmHg·s·mL^−1^, less flow crosses PDA to aorta. In this situation, since the pulmonary pressure is higher than the aortic pressure, there would be more systemic flow than pulmonary flow. The pressure curves for this stage are shown in Figure 11. In this situation, the condition becomes extremely dangerous. All the flow goes to the lungs and there is a reverse pressure gradient across the PDA such that the pressure in the PA is less than the pressure in the aortic arch and there is no flow going to the vital organs. Pressure builds in the RA because of the extra small pulmonary flow and death ensues.

Norwood procedure is performed to create a neoaorta from the common pulmonary artery as the cardiac outflow track. A synthetic shunt of constant diameter is placed to stabilize the pulmonary blood flow and the PDA is legated.

The constant diameter of the shunt limits the amount of flow to the low resistance PA, thereby stabilizing *Qp*/*Qs*, a model which has been presented in [20].

Three parameters,*R*_ASD_, *R*_PA_, and *R*_PDA_, were chosen and were varied over a range of physiological resistance, and the resulting changes in the hemodynamic parameters were analyzed. Varying ASD resistance (*R*_ASD_) corresponds to varying magnitudes of the septal defect. Varying the pulmonary artery resistance (*R*_PA_) corresponds to the increasing age of the baby. Finally, the last parameter varied is the resistance within the PDA (*R*_PDA_). This parameter can be controlled by an infusion of prostaglandin to prevent PDA closure.

**Figure 12:**
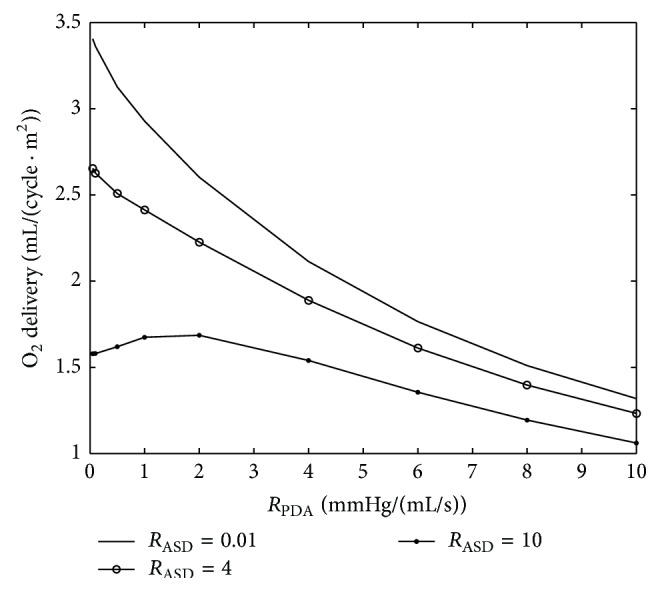
Systemic oxygen delivery. Oxygen delivery is calculated by multiplication of system flow and arterial oxygen concentration. The results are consistent with what is presented in [20].

**Figure 13:**
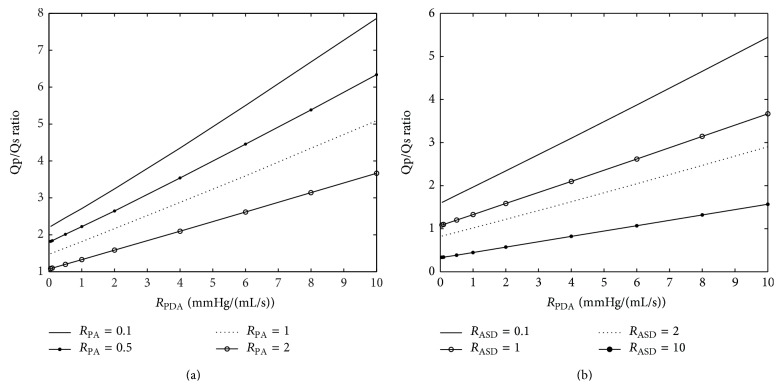
Variation of *Qp*/*Qs* by changing the most important model parameters. Values around one are normal.

**Figure 14:**
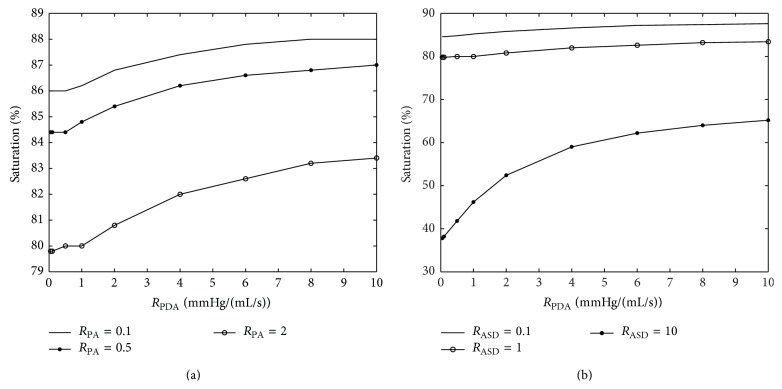
Variation of arterial oxygen saturation by changing the most important model parameters.

**Figure 15:**
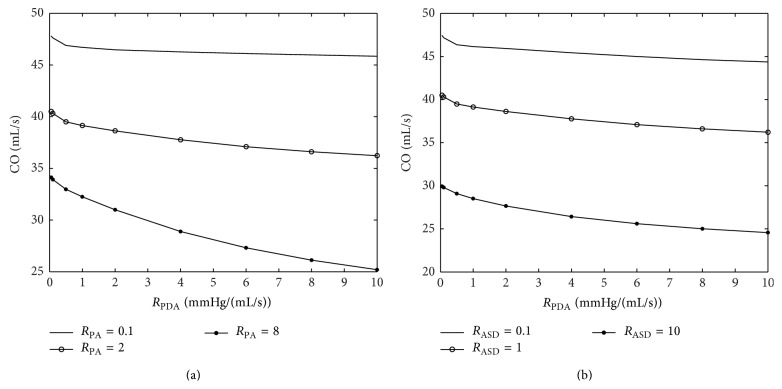
Variation of cardiac output by changing the *R*_ASD_, *R*_PA_, and *R*_PDA_.

Two parameters are varied in each plot to study the effects of the parametric variation on systemic hemodynamics. The graphs of important hemodynamic parameters such as cardiac output, pulmonary to systemic flow ratio, and oxygen and carbon dioxide partial pressures are presented (Figures 12–15). Systemic oxygen delivery as a function of increasing *R*_PDA_ is shown in Figure 12. We can observe from this plot that oxygen delivery will decrease with increase of both *R*_ASD_ and *R*_PDA_. Although this is a fairly obvious fact, the interesting finding is that in the case of very high *R*_ASD_ the oxygen delivery is not monotonically decreasing, and it has a maximum for some intermediate values of *R*_PDA_. We can infer that in the case of neonates with very high *R*_ASD_ an increase in *R*_PDA_ to some extent could be helpful.

As can be observed from Figures 13(a) and 13(b), the pulmonary-to-systemic flow ratio (*Qp*/*Qs*) changes with the change in the model parameters. As *R*_ASD_ increases, this ratio decreases because the pulmonary flow keeps on decreasing; this is because, at steady state, all the pulmonary flow passes through the ASD, and an increase of *R*_ASD_ leads to a decrease in the flow. As *R*_PDA_ increases, the systemic flow decreases since all the systemic flow passes through ductus arteriosus which leads to an increase in the *Qp*/*Qs* ratio.

A *Qp*/*Qs* ratio around one is optimal for the body [28] and hence a suitable area for the flow can be selected from these graphs accordingly. Considering the parameters that have to be changed, such a point can be reached. The direction of blood flow depends upon the resistance, and hence, in the case of HLHS, blood follows the path of least resistance. In high *R*_PA_ resistances in constant *R*_PDA_, the ratio decreases because, due to high pulmonary resistance, the amount of pulmonary flow decreases and systemic flow increases leading to a decrease in the *Qp*/*Qs* ratio. Plots show that in some combination of PA, ASD, and PDA resistances the optimal *Qp*/*Qs* can never be achieved. For example, if *R*_PA_ = 0.1 mmHg·s·mL^−1^, the optimal *Qp*/*Qs* ratio is unreachable.

As shown in Figures 14(a) and 14(b), as *R*_PDA_ increases, the arterial O_2_ saturation also increases and becomes nearly constant at high values of the resistance; however, it decreases monotonously with *R*_ASD_. The decrease with *R*_ASD_ becomes more profound at lower values of *R*_PDA_. This trend is also observed with *R*_PA_. For constant *R*_PA_, systemic arterial O_2_ concentration increases with increasing *R*_PDA_.

As *R*_PDA_ increases, cardiac output (sum of pulmonary and systemic flows) decreases slowly as the systemic flow keeps on decreasing and this counters the effect of the increasing pulmonary flow as shown in Figures 15(a) and 15(b). As *R*_ASD_ increases, cardiac output decreases significantly due to a rapid reduction in the pulmonary flow. Hence, it can be deduced that the sensitivity of the pulmonary flow to *R*_ASD_ is very high. A similar trend can be observed with *R*_PA_ playing the same role as that of *R*_ASD_.

**Figure 16:**
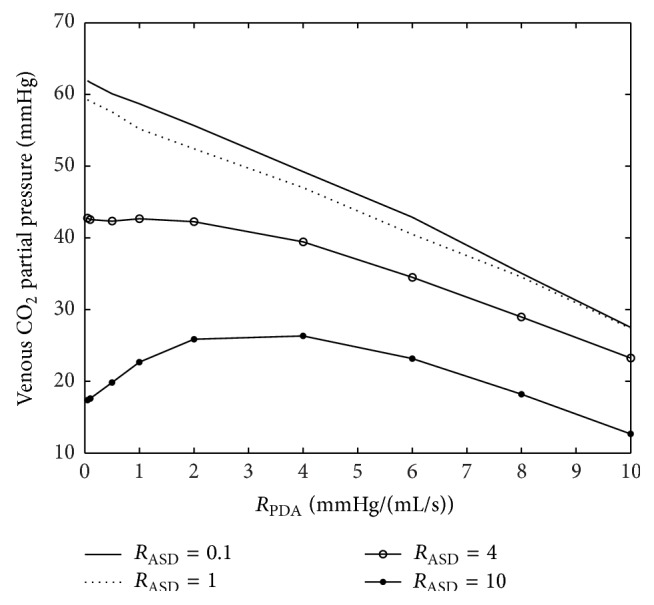
Variation of *P*_CO_2__ for constant *R*_PA_ = 0.1 mmHg·s·mL^−1^ and changing *R*_PDA_ and *R*_ASD_. Previous finding underlined the importance of *P*_CO_2__ as a risk factor for the occurrence of PVL.

Figure 16 shows a plot of *P*_CO_2__ (partial pressure of carbon dioxide) for constant *R*_PA_ = 0.1 mmHg·s·mL^−1^ and varying values of *R*_PDA_ and *R*_ASD_. We focus on *P*_CO_2__ since our previous studies based on computational intelligence (CI) techniques have shown that 43 mmHg *P*_CO_2__ is an important threshold for predicting PVL. By inclusion of *P*_CO_2__ in our model we attempt to find a reason for this interesting but somewhat inexplicable result of the CI technique. Our results show that all values of *P*_CO_2__ are less than 43 mmHg for *R*_PA_ = 0.1 mmHg·s·mL^−1^ and *R*_ASD_ ≥ 4 mmHg·s·mL^−1^. Considering the fact that *P*_CO_2__ to some extent is measureable and controllable post- and preoperatively, this finding could well lead to a valuable insight.

## 4. Conclusion

The goal of this paper has been to develop a lumped parameter model of HLHS, a fairly common congenital heart disease. The need for this physical modeling comes from our ultimate goal to predict and prevent one of the consequences of this abnormal circulation, namely, a form of brain injury called periventricular leukomalacia (PVL).

The exact causes of PVL are not well understood and in this paper our focus has been on understanding how some critical parameters such as flow resistances at the atrial septal defect or patent ductus arteriosus might alter systemic flow and systemic oxygen delivery, which could be reasonably expected to have a causal relationship to PVL occurrence.

In this paper, we have compared the results of HLHS circulation (preoperative condition) to Norwood circulation (postoperative condition). Results show the manner and extent of changes in the pressure and blood flow in different parts of the body. In reporting the modeling results, we have mostly focused on the pulmonary to systemic flow ratio (*Qp*/*Qs*), O_2_ delivery, and O_2_ saturation because we believe that these parameters are important risk parameters of the PVL occurrence. By use of the model, the following points were demonstrated or confirmed.

Different sizes of the PDA and the ASD and different resistances of the PDA and the ASD will result in changing the regulation of pulmonary and systemic flow and hence these resistances play a critical part in our goal to predict the occurrence of the PVL.

The direction of blood flow depends upon the PA resistance and that of the PDA resistance. Increase in the PDA resistance leads to blood flowing where the resistance is the least. This results in an increased *Qp*/*Qs* ratio. With an increase in the PDA resistance a greater portion of the cardiac output goes into the lungs. This will lead to a systemic hypoperfusion and poor O_2_ delivery. This result is consistent with the results presented in [29].

By comparing the slope in cardiac output and pulmonary to systemic flow variation plots, one could observe that the rates of change (slopes) are higher for variation of PA resistance in comparison with the variation of ASD resistance. This means that the PA resistance is more influential on the CO and pulmonary to systemic flow ratio than the ASD resistance. This suggests that the PA resistance is more directly involved in the determination of the *Qp*/*Qs* ratio and the cardiac output.

The optimal O_2_ delivery is achieved when balanced pulmonary and systemic perfusion is established, namely, when *Qp*/*Qs* is around 1. Our results show that, for some values of *R*_PA_ and *R*_ASD_, the optimal *Qp*/*Qs* can never be achieved. Since insufficient O_2_ delivery is considered to be a potential cause for PVL, it seems reasonable to infer that these values of *R*_ASD_ and *R*_PA_ lead to high risks of PVL.

It is known that the correlation between venous O_2_ saturation and O_2_ delivery is better than the correlation between arterial O_2_ saturation and O_2_ delivery. However, since measurement of the mixed venous saturation in clinical practice remains difficult, we just considered the case of arterial oxygen saturation [30] in this study.

The developed model also has some other interesting applications. For example, with some minor modifications to cover the partly underdeveloped left heart, the proposed model could form a good foundation for the analysis of newborns with underdeveloped left ventricles and model based design of left ventricular assist devices.

Given that the exact causes of PVL are still not clearly understood, the main goal of this paper has been to develop a sound mathematical model to investigate HLHS, a severe congenital deformation which has the highest correlation with the occurrence of PVL as suggested by preliminary postoperative data collected from Children's Hospital of Philadelphia (CHOP). Also, the paper aims to provide a tool to better understand the main factors that drive the HLHS physiology by a suitable variation of physiological parameters.

Our previous computational intelligence based findings [5, 7, 10] suggest some important factors in the occurrence of PVL; however, since the exact physiological reasons and causes of those findings are not clear, the current model has been developed to discover cases that will lead to the PVL situation and to analyze them. We hope that the developed model could open the door for further investigation of the HLHS syndrome and also to its connection with PVL. The postoperative results are consistent with what is reported in [20] which makes this model potentially valuable for clinical use.

Some of the limitations of the current work are as follows.

- The effect of respiration and regulatory mechanisms such as HR baroreflex and total peripheral resistance baroreflex on the cardiopulmonary performance is not considered in building the model.
- The values of the components of the analog electrical circuit are estimated and there is still no direct, reliable, and safe way to measure them with high confidence.
- The results of the model should be validated clinically. A parameter adaptation algorithm for patient specific modeling should be developed and implemented on the patient specific data. The correlation between the PVL occurrence and different values for ASD, PA, and PDA resistances should be calculated for the aim of PVL occurrence prediction.

To improve the model further and to add more verisimilitude with reality, it is important to include other factors into the system such as autoregulation and to investigate its effects. Our current work is continuing on this important problem and will focus on addressing some of the above limitations.

Finally we would like to comment about the general utility of the procedures developed in this paper. With all the obvious limitations, the method of constructing a mathematical model pursued in this paper is still appealing because it permits the change of parameters very easily to understand their influence on the usual key clinical outputs such as *Qp*/*Qs*. Such models exist for other conditions but none exist for PVL based on preoperative conditions. By use of a parametric analysis on computer models we start to acquire the very useful ability to predict pre- and postoperative conditions for specific patients and also to develop patient specific models to investigate physiological states of individual patients. That said, we would like to reiterate the limitations of the current model and hope to develop and inspire development of improved models with appropriate corroboration.

## Figures and Tables

**Table 1:** Heart model parameters.

Parameters	Values
Right atrium	
*E*_1_, RA	7.35 mmHg/mL
*P*_0_, RA	0.17 mmHg
*K*_*e*_, RA	0.484 mL^−1^
*V*_*u*_, RA	1 mL

Left atrium	
*E*_1_, LA	7.35 mmHg/mL
*P*_0_, LA	0.17 mmHg
*K*_*e*_, LA	0.484 mL^−1^
*V*_*u*_, LA	1 mL

Right ventricle	
*E*_1_, RV	8.5 mmHg/mL
*E*_2_, RV	−0.042 mmHg/mL^2^
*P*_0_, RV	0.9 mmHg
*K*_*e*_, RV	0.062 mL^−1^
*V*_*u*_, RV	4 mL

ASD	
*R*_ASD_	0.1 mmHg·s·mL^−1^

Tricuspid valve	
*K*_Tric_	0.0006 mmHg·s^2^·mL^−1^

Pulmonary valve	
*K*_PV_	0.0008 mmHg·s^2^·mL^−1^

Activation function	
*K*_*e*_ (ventricle)	51
*K*_*e*_ (atrium)	75
*K*_*r*_	55
*t*_*s*_ (ventricle)	0.193 s
*t*_*s*_ (atrium)	0.173 s
*α*_max_ (ventricle)	150
*α*_max_ (atrium)	130

**Table 2 tab2:** Circulation parameters.

Parameters	Values
Pulmonary circulation	

PA	
*R*	2 mmHg·s·mL^−1^
*L*	0.00412 mmHg·s^2^·mL^−1^
*C*	0.27410 mL/mmHg

PAB	
*R*	0.41688 mmHg·s·mL^−1^
*C*	0.04078 mL/mmHg

PVB	
*R*	0.01097 mmHg·s·mL^−1^
*L*	0.00218 mmHg·s^2^·mL^−1^
*C*	0.88750 mL/mmHg

Systemic circulation	

DA	
*R*	0.19883 mmHg·s·mL^−1^
*L*	0.00287 mmHg·s^2^·mL^−1^
*C*	0.06118 mL/mmHg

SAB	
*R*	3.51120 mmHg·s·mL^−1^
*L*	0.00535 mmHg·s^2^·mL^−1^
*C*	0.44296 mL/mmHg

SVB	
*R*	0.32255 mmHg·s·mL^−1^
*C*	0.31030 mL/mmHg

SLV	
*R*	0.08265 mmHg·s·mL^−1^
*C*	4.07890 mL/mmHg

Patent ductus arteriosus	
*R*	1 mmHg·s·mL^−1^

**Table 3:** Blood gas diffusion parameters.

Parameters	Values
Oxygen	
*D*_*L*O_2__	180 mL/min
Γ	0.00003 (mL·mL^−1^mmHg^−1^)
*B*	0.2213 (mL·mL^−1^mmHg^−1^)
*N*	2.7
*P*_avO_2__	100 mmHg

Carbon dioxide	
*D*_*L*CO_2__	450 mL/min
*V*_cap_	3.5 mL
*P*_avCO_2__	41 mmHg
